# Antiprotozoal Activity against *Entamoeba histolytica* of Plants Used in Northeast Mexican Traditional Medicine. Bioactive Compounds from *Lippia graveolens* and *Ruta chalepensis*

**DOI:** 10.3390/molecules191221044

**Published:** 2014-12-15

**Authors:** Ramiro Quintanilla-Licea, Benito David Mata-Cárdenas, Javier Vargas-Villarreal, Aldo Fabio Bazaldúa-Rodríguez, Isvar Kavim Ángeles-Hernández, Jesús Norberto Garza-González, Magda Elizabeth Hernández-García

**Affiliations:** 1 Universidad Autónoma de Nuevo León, UANL, Facultad de Ciencias Biológicas, Av. Universidad S/N, Cd. Universitaria, San Nicolás de los Garza, C.P. 66451 Nuevo León, Mexico; E-Mails: abazaldua5@hotmail.com (A.F.B.-R.); isvark@gmail.com (I.K.Á.-H); 2 Universidad Autónoma de Nuevo León, UANL, Facultad de Ciencias Químicas, Av. Universidad S/N, Cd. Universitaria, San Nicolás de los Garza, C.P. 66451 Nuevo León, Mexico; E-Mail: benitodavidm@yahoo.com.mx; 3 Laboratorio de Bioquímica y Biología Celular, Centro de Investigaciones Biomédicas del Noreste (CIBIN), Dos de abril esquina con San Luis Potosí, C.P. 64720 Monterrey, Mexico; E-Mails: jvargas147@yahoo.com.mx (J.V.-V.); jesusnorbertogarza@hotmail.com (J.N.G.-G.); magdaehg@hotmail.com (M.E.H.-G.)

**Keywords:** neglected diseases, amoebiasis, Mexican medicinal plants, bioguided isolation, carvacrol, chalepensin, antiprotozoal agents, 1D- and 2D-NMR data

## Abstract

Amoebiasis caused by *Entamoeba histolytica* is associated with high morbidity and mortality is becoming a major public health problem worldwide, especially in developing countries. Because of the side-effects and the resistance that pathogenic protozoa build against the standard antiparasitic drugs, e.g., metronidazole, much recent attention has been paid to plants used in traditional medicine around the world in order to find new antiprotozoal agents. We collected 32 plants used in Northeast Mexican traditional medicine and the methanolic extracts of these species were screened for antiprotozoal activity against *E. histolytica* trophozoites using *in vitro* tests. Only 18 extracts showed a significant inhibiting activity and among them six plant extracts showed more than 80% growth inhibition against *E. histolytica* at a concentration of 150 µg/mL and the IC_50_ values of these extracts were determined. *Lippia graveolens* Kunth and *Ruta chalepensis* Pers. showed the more significant antiprotozoal activity (91.54% and 90.50% growth inhibition at a concentration of 150 µg/mL with IC_50_ values of 59.14 and 60.07 µg/mL, respectively). Bioassay-guided fractionation of the methanolic extracts from these two plants afforded carvacrol (**1**) and chalepensin (**2**), respectively, as bioactive compounds with antiprotozoal activity.

## 1. Introduction

An estimated billion people are infected with one or more neglected tropical diseases [1,2,3,4]. Amoebiasis caused by *Entamoeba histolytica*, a protozoan of the family Endomoebidae [5,6], is associated with high morbidity and mortality and has become a major public health problem worldwide [7] and is therefore considered as the third parasitosis of medical importance after malaria and schistosomiasis [8]. *E. histolytica* is still endemic in tropical and sub-tropical regions, causing a high incidence of infections in developing countries in Latin America, Asia and Africa [9], where poor sanitary conditions, population explosion and inadequate control of reservoirs intensify the development of these infections [10]. The amoebiasis is prevalent throughout the developing nations with tropical ecosystems, at times reaching a prevalence of 50% of the general population and is estimated to cause more than 100,000 deaths per year [11,12]. Symptomatic patients typically may develop abdominal pain and tenderness, diarrhea, and bloody stools, but the disease may spread to the liver and other organs resulting in death [13,14].

Currently metronidazole is the therapeutic drug of choice for the treatment of amoebiasis [15], but is experiencing drug resistance by *E. histolytica* [16,17], resulting in the need for increased doses to overcome the infection [18] and thus causing unpleasant side-effects, such as headache, nausea, dry mouth, and a metallic taste, as well as neurotoxicity [19,20]. Owing to these undesired side effects and taking into account the development of resistant strains of *E. histolytica* against metronidazole, new, more effective and safer antiprotozoal agents are urgently required [20,21]. Natural products have proved to be an important source of lead compounds in the development of new drugs and artemisinin, quinine and licochalcone A are all examples of plant-derived products with antiparasitic activity [22,23]. Screening natural products provides the chance to discover new molecules of unique structure with high activity and selectivity [24].

Thus, with the purpose of searching for new antiprotozoal agents, 32 medicinal plants used in Northeast Mexican traditional medicine [25,26] were selected to evaluate the activity of their methanol crude extracts against *E. histolytica* trophozoites. The selection of the species was mainly based on a follow up of ethnobotanical uses for the treatment or relief of symptoms related with parasitic infections.

## 2. Results and Discussion

### 2.1. In Vitro Susceptibility Assays of Plant Extracts

In this work, we report the antiprotozoal activity of 32 crude methanolic extracts derived from plants used in Northeast Mexico for the treatment of gastrointestinal disorders. The yields after Soxhlet extraction of each plant are shown in Table 1.

**Table 1:** Soxhlet extraction of medicinal plants used in Northeast Mexico investigated for antiprotozoal activity.

Scientific Name	Family	Voucher Specimen	Part Used ^a^	Yield (%^w^/_w_) ^b^
***Agave lechugilla*** **Torr**	Agavaceae	025529	L	16.5
***Amphipterygium adstringens*** **Standley**	Julianaceae	025530	B	18.8
***Apium graveolens*** **Linnaeus**	*Apiaceae*	025531	S	5.7
***Arctostaphylos pungens*** **Kunth**	Ericaceae	025532	FR	23.3
***Artemisia mexicana*** **Willd**	Asteraceae	025533	L	15.3
***Bougainvillea spectabilis*** **Willd**	Nyctaginaceae	025535	F	24.5
***Capsicum annuum*** **Linnaeus**	Solanaceae	025536	FR	37.9
***Castela texana*** **Torr & Grey**	Simaroubaceae	025538	L	14.0
***Cecropia obtusifolia*** **Bertol**	Cecropiaceae	025539	AP	12.6
***Coriandrum sativum*** **Linnaeus**	Apiaceae	025540	L	31.8
***Cyclolepis genistoides*** **Don**	Asteraceae	025541	L	16.5
***Cymbopogon citrates*** **Stapf**	Poaceae	025542	L	17.6
***Eryngium heterophyllum*** **Engelm**	Apiaceae	025544	A	15.3
***Eucalyptus globulus*** **Labill**	Myrtaceae	025545	L	10.8
***Foeniculum vulgare*** **Miller P.**	Apiaceae	025546	L	12.4
***Gnaphalium oxyphyllum*** **DC**	Asteraceae	025572	L	11.2
***Gymnosperma glutinosum*** **Spreng**	Asteraceae	025547	AP	39.9
***Haematoxylon brasiletto*** **Karsten**	Leguminosae	025548	L	18.8
***Heterotheca inuloides*** **Cass**	Asteraceae	025549	AP	16.3
***Hibiscus sabdariffa*** **Linnaeus**	Malvaceae	025550	F	44.0
***Juglans mollis*** **Engelm**	Juglandaceae	025551	L	6.3
***Lippia graveolens*** **Kunth**	Verbenaceae	025554	AP	41.0
***Marrubium vulgare*** **Linnaeus**	Lamiaceae	025555	AP	15.6
***Melissa officinalis*** ** Linnaeus**	Lamiaceae	025557	L	17.9
***Mentha spicata*** **Crantz**	Lamiaceae	025558	L	18.9
***Ocimum basilicum*** **Linnaeus**	Lamiaceae	025559	L	18.5
***Opuntia ficus-indica*** **Linnaeus**	Cactaceae	025560	CL	17.0
***Persea Americana*** **Mill**	Lauraceae	025563	L	21.2
***Ruta chalepensis*** **Pers**	Rutaceae	025579	AP	12.7
***Schinus molle*** ** Linnaeus**	Anacardiaceae	025567	AP	15.9
***Syzygium aromaticum*** **Linnaeus**	*Myrtaceae*	025569	F ^c^	39.9
***Tilia platyphyllos*** **Scopoli**	Tiliaceae	025570	F	11.8

^a^ Plant part extracted: AP, aerial parts; B, barks; CL, cladodes; F, flowers; FR, fruits; L, leaves; S, seeds; ^b^ Percentage based on dried plant material; ^c^ Not opened flower.

*In vitro* susceptibility assays were performed for each crude extract. Table 2 summarizes the antiprotozoal activity on *Entamoeba histolytica* of the plant extracts and the control drug (metronidazole). Extracts from 18 out of the 32 samples tested showed significant growth inhibition of *E. histolytica* with percentage values ranging from 24.65 to 91.54 at a concentration of 150 µg/mL. The remaining 12 plants showed absolutely no activity. *Lippia graveolens* Kunth, *Ruta chalepensis* Pers, *Capsicum annuum* Linnaeus, *Opuntia ficus-indica* Linnaeus, *Haematoxylon brasiletto* Karsten and *Schinus* molle Linnaeus displayed more than 80% growth inhibition against *E. histolytica* at a concentration of 150 µg/mL, with IC_50_ values ranging from 32.45 to 98.75 µg/mL, far less effective than metronidazole (IC_50_ 0.205 µg/mL), but these IC_50_ values are suitable as selection criterion for further investigation of these plant extracts as source of potential antiprotozoal agents [10].

**Table 2:** Antiprotozoal activity against *Entamoeba histolytica* of methanolic extracts ^a^ from selected plants.

Plant Specimen	% Growth Inhibition ^b^	IC_50_ of Crude Extract (µg/mL) ^b^
***Lippia graveolens*** **Kunth**	91.54	59.14
***Ruta chalepensis*** **Pers**	90.50	60.07
***Capsicum annuum*** **Linnaeus**	87.87	98.75
***Opuntia ficus-indica*** **Linnaeus**	87.47	70.33
***Haematoxylon brasiletto*** ** Karsten**	84.84	96.38
***Schinus molle*** **Linnaeus**	81.79	32.45
***Melissa officinalis*** **Linnaeus**	76.95	^c^
***Castela texana*** **Torr & Grey**	73.82	^c^
***Cyclolepis genistoides*** **Don**	73.80	^c^
***Juglans mollis*** **Engelm**	71.87	^c^
***Agave lechugilla*** **Torr**	69.66	^c^
***Mentha spicata*** **Crantz**	65.72	^c^
***Tilia platyphyllos*** **Scopoli**	65.00	^c^
***Gymnosperma glutinosum*** **Spreng**	63.80	^c^
***Gnaphalium oxyphyllum*** **DC**	42.15	^c^
***Apium graveolens*** **Linnaeus**	29.03	^c^
***Cecropia obtusifolia*** **Bertol**	29.00	^c^
***Persea americana*** **Mill**	24.65	^c^

^a^ Adjusted to a concentration of 150 µg/mL; ^b^ Metronidazole 100% growth inhibition (IC_50_ = 0.205 µg/mL); ^c^ IC_50_ was not determined.

It is important to point out that the antiprotozoal activity of seven plants chosen for this work has been previously reported. However, we decided to evaluate these species again because the antiprotozoal activity was tested with different parasites or extracts. From *Lippia graveolens* the biological activity mainly of its essential oils against *Giardia lamblia* [27,28,29] and *Leishmania infantum* [30] has been reported. There have been previous reports describing the activity of methanolic extracts (macerated at room temperature) of *Artemisia mexicana*, *Ocimum basilicum*, *Ruta chalepensis* and *Schinus molle* against trophozoites of *E. histolytica* and *G. lamblia* [10], reporting IC_50_ values for these plants of 82.2, 41.7, 61.9 and 82.4 µg/mL, respectively, against *E. histolytica*. Although we observed a similar IC_50_ to the one reported by Calzada [10] for *R. chalepensis*, we did not notice any activity for *A. mexicana* and *O. basilicum*, but observed higher activity for *S. molle*, which could be explained by the fact that the plant material was provided by different regional suppliers. In addition, *R. chalepensis* also showed activity against *L. infantum* and *L. major* [31], and *S. molle* also showed activity against *Plasmodium falciparum*, *Trypanosoma brucei*, *T. cruzi*, and *L. infantum* [32]. *Melissa officinalis* has demonstrated biological activity reported against cysts and trophozoites of *Acanthamoeba castellanii* [33]. Some reports on *Castela texana* revealed that the ethanolic extract of aerial parts and the methanolic extract from roots have relevant amebicide activity [34,35,36]. We also found good amebicide activity for this plant (73.82% growth inhibition at a concentration of 150 µg/mL) by using leaves for the preparation of a methanolic extract.

To our knowledge, this is the first report of antiprotozoal activity against *Entamoeba histolytica* of extracts from *Agave lechugilla* Torr, *Apium graveolens* Linnaeus, *Capsicum annuum* Linnaeus, *Cecropia obtusifolia* Bertol, *Cyclolepis genistoides* Don, *Gnaphalium oxyphyllum* DC, *Gymnosperma glutinosum* Spreng, *Haematoxylon brasiletto* Karsten, *Juglans mollis* Engelm, *Melissa officinalis* Linnaeus, *Mentha spicata* Crantz, *Opuntia ficus-indica* Linnaeus, *Persea americana* Mill and *Tilia platyphyllos* Scopoli.

### 2.2. Phytochemistry of Bioactive Plants against Entamoeba histolytica

There are few reports relating to the antiprotozoal activity of pure compounds isolated from the plants included in this study. Some flavonoids occurring in *L. graveolens*, e.g., apigenin, (−)-epigallocatechin, galangin, kaempferol, narigenin, pinocembrin and quercetin [37,38] showed biological activity against *E. histolytica* and *G. lamblia* [39]. Chaparrin (a simaroubolidane), presenting 100% growth inhibition against *E. histolytica* at a concentration of 100 µg/mL, was isolated from the roots of *Castela texana* [34]. The isolated quassinoid from *C. texana*, 11-*O-trans-p-*coumaroyl amarolide also presented antimalarial activity [40].

For the other bioactive plants no reports concerning antiprotozoal activity were found, but remarkable phytochemical research has been done in order to isolate the bioactive compounds responsible for different biological activities. Phytochemical studies showed the presence of steroidal saponins with anti-inflammatory properties in *Agave lechugilla* [41,42,43]. From *Apium graveolens* β-selinene and sedanolide with mosquitocidal, nematicidal and antifungal activity have been isolated [44,45]. Furthermore, sesquiterpenoid, phtalide, aromatic and lignan glucosides were isolated from *A*. *graveolens* [46,47]. Several capsaicinoids with antioxidant activity [48] as well as sesquiterpenoids with cytotoxic activity [49] have been isolated from *Capsicum annuum*. In addition, various phenylpropanoids with inhibitory action against *Listeria monocytogenes* were isolated from *C*. *annuum* [50]. A phytochemical study of Andrade-Cetto *et al.* [51] confirmed the hypoglycemic effect of chlorogenic acid and isoorientin, main components of *Cecropia obtusifolia*. Several triterpenes and sesquiterpene lactones have been isolated from *Cyclolepis genistoides* [52]. Moreover, oleanolic acid and deacylcynaropicrin, compounds with anti-inflammatory properties, were isolated from *C*. *genistoides* [53]. From *Gnaphalium oxyphyllum* several diterpenoids, acetylenic compounds, and carotenoids with antimicrobial activity have been isolated [54]. It has been reported that *Gymnosperma glutinosum* contains flavonoids and diterpenes with antimicrobial, antifungal and cytotoxic activities [25,55,56]. From *Haematoxylon brasiletto* the neoflavonoids hematoxylin and brazilin as well as caffeic acid, gallic acid and 4-hydroxycinammic acid, all of them compounds with antimicrobial activity, have been isolated [57]. Several monoterpene hydrocarbons, caffeate oligomers, flavonoids and terpenoids have been identified in *Melissa officinalis* [58,59]. Many terpenes isolated from the essential oils of *Mentha spicata* possess a wide spectrum of biological activity against many pathogenic bacteria, fungi and some protozoa [60]. From *Opuntia ficus-indica* the alkaloids indicaxanthin and neobetanin as well as various flavonoids have been isolated [61]. (*E,Z,Z*)-1-Acetoxy-2-hydroxy-4-oxo-heneicosa-5,12,15-triene was isolated from avocado, *Persea americana*; it inhibited spore germination of the fungal pathogen *Colletotrichum gloeosporioides* [62]. Furthermore, persenone A and B with good activity as inhibitors of nitric oxide and superoxide generation were isolated from *Persea Americana* [63]. Several furanocoumarins and quinoline alkaloids with antimicrobial [64] and larvicidal [65] activities have been isolated from *Ruta chalepensis*. Likewise several sesquiterpenoids, triterpenoids and flavonoids have been isolated from *Schinus molle* [66]. From *Tilia platyphyllos* some monoterpenic hydrocarbons and alcohols as well as flavonoids have been identified [67,68,69].

Neither chemical nor biological reports concerning antiprotozoal activity of *Juglans mollis* could be found in the literature, but from the related plant *Juglans regia* oleic, linoleic, α-linoleic and ellagic acid as well as the flavonoid juglamin have been isolated [70,71].

Bioassay-guided fractionation of the bioactive extracts from the plants included in this study will be carried out in order to isolate pure compounds related to their antiprotozoal activity. *Lippia graveolens* Kunth and *Ruta chalepensis* Pers showed the most significant antiprotozoal activity (91.50 and 90.50% growth inhibition at a concentration of 150 µg/mL with IC_50_ values of 59.14 and 60.07 µg/mL, respectively), therefore these plants were the first choice for subsequent work on the isolation of their active constituents.

### 2.3. Isolation and Structure Elucidation of Compounds with Antiprotozoal Activity

**Figure 1:**
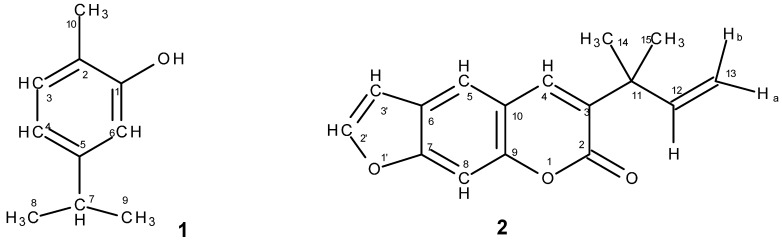
Structure of carvacrol (**1**) and chalepensin (**2**).

Bioassay-guided fractionation of the methanolic extract from *L. graveolens* rendered carvacrol (**1**) with 95%–98% inhibition against *E. histolytica* at a concentration of 150 µg/mL (IC_50_ 44.3 µg/mL) and from the methanolic extract of *R. chalepensis* chalepensin (**2**) with 98.4% inhibition at a concentration of 150 µg/mL (IC_50_ 45.95 µg/mL) was recovered. Identification of the isolated compounds was based on spectroscopic/spectrometric analyses (IR, ^1^H- and ^13^C-NMR; MS) and comparison with literature data. The corresponding chemical structures are shown in Figure 1.

#### 2.3.1. Carvacrol from *Lippia graveolens*

Preliminary fractionation of the methanolic extract of *Lippia graveolens* by extraction with *n*-hexane led, after evaporation of solvent, to a residue with good activity against *E. histolytica* (90.9% growth inhibition at a concentration of 150 µg/mL). Chromatography of this hexane residue over a silica gel column led to the isolation of carvacrol (**1**) as a colorless oil. Spectroscopic data of **1** were in concordance with literature values [72,73,74].

The essential oil of *Lippia graveolens* contains many monoterpenes, sesquiterpenes and phenolic terpenes among which carvacrol and thymol are the most common components [75,76,77] and their average abundance establishes the chemotype that can be assigned to *L. graveolens* varieties [78,79,80].

Due to its acidic and hydrophobic nature, carvacrol tends to damage biological systems and for that reason is responsible for affecting a wide range of microorganisms, including bacteria, fungi, yeast and parasites [81,82,83,84,85]. It also has been proposed as a therapeutic agent against some cancer cell lines due to its activity as an antiproliferative compound [86,87], DNA synthesis inhibitor [88] and by triggering apoptosis [89,90].

The monoterpenic phenol exerts a bactericidal effect against many foodborne bacteria responsible for gastrointestinal disorders, such as *Listeria monocytogenes*, *Escherichia coli* [91], *Salmonella enterica* ser. Enteritidis [92], *Bacillus subtillis*, *Salmonella typhimurium*, *Escherichia coli* [93,94], *Shigella sonnei*, *S. flexeri* [95] and it has been considered useful to inhibit the growth of microorganisms responsible for respiratory disorders like *Staphyloccocus aureus*, *Staphyloccocus aureus MSRA*, *Streptococcus pneumonie*, *Klebsiella pneumonie*, *Pseudomonas auroginosa* [85]. Carvacrol is also able to inhibit the enterotoxin production from *Bacillus cereus*, which generates abdominal pain and diarrhea [96].

Important toxigenic and pathogenic filamentous fungi, some causative of serious human mycoses, have been subjected to carvacrol exposure and antifungal properties were found for this compound against *Aspergillus niger*, *A. flavus*, *A. fumigatus*, *Penicillum digitatum*, *P. brevicompactum*, *P. expansum and Fusarium spp*. [97,98,99]. Carvacrol has also shown strong fungicidal effects against many clinical isolates from *Candida spp*. and *Cryptococcus spp*. [97,100].

Furthermore, carvacrol was tested against many tropical parasites responsible for serious human diseases. Thus, the compound was evaluated for its trypanocidal activity against *Trypanosoma cruzi* and *T. brucei* rhodesiense, being very effective in both cases, with IC_50_ values under 30 µg/mL [101,102,103], but noteworthily an important inhibition effect was observed on the epimastigote form in *T. cruzi* isolates (IC_50_ 3.0 µg/mL). Following this topic, carvacrol was evaluated against visceral parasites of the genus *Leishmania* and the results demonstrated an important effectiveness range over the promastigote form of *Leishmania chagasi* with IC_50_ values between 2.3 to 28 µg/mL [103,104]. A weak activity was observed on *Leishmania donovani* with an IC_50_ value of 17.8 mg/mL, compared with the reference drug miltefosine (IC_50_ 0.14 mg/mL). In addition, antimalarial activity against *Plasmodium falciparum* was tested, obtaining a very significant IC_50_ value of 7.9 mg/mL [85].

Prior to our investigation, amebicide evaluation of carvacrol had not been carried out on *E. histolytica*, but one clinical study was performed on 14 adult patients, whose stools tested positive for enteric parasites such as *Entamoeba hartmanni*, *Endolimax nana* and *Blastocystis hominis*. The patients were supplemented with an essential oil rich in carvacrol from *Origanum vulgare* and after 6 weeks of treatment total disappearance of *E. hartmanni* and *E. nana* was observed in all infected patient cases. *B. hominis* was not detected in five cases [105]. Nevertheless this is the first report about the antiprotozoal activity of carvacrol against *Entamoeba histolytica*.

#### 2.3.2. Chalepensin from *Ruta chalepensis*

The bioguided fractionation of the methanolic extract of *Ruta chalepensis* by partition between methanol and *n*-hexane followed by chromatography of the hexane residue (84.66% growth inhibition against *E.* *histolytica* at a concentration of 150 µg/mL) over a silica gel column afforded chalepensin (**2**) as colorless needles with a melting point of 75 °C. The spectroscopic data were identical with those reported for chalepensin [106,107,108,109,110,111,112,113], but we now report the complete, unambiguous assignment of the ^13^C-NMR spectrum of chalepensin as the hydrogen and carbon connectivities in **2** were deduced from ^1^H-^1^H COSY, NOESY, HSQC and HMBC spectra.

Chalepensin has been isolated from several herbs, especially those of the Rutaceae family, including *Ruta chalepensis* [114,115,116,117,118,119,120]. Chalepensin has strong allelochemical and phytotoxic activity [121,122,123]. Moreover, chalepensin has a variety of pharmacological effects, including a significant anti-fertility activity [124], antiplatelet aggregation activity [112], cytotoxic effects on some human carcinoma cell lines (breast MCF-7; colon HT-29; kidney A-498; lung A-549; pancreatic PACA-2; prostate PC-3) [122], and further antiproliferative activity against human gastric adenocarcinoma (MK-1), human uterus carcinoma (HeLa) and murine melanoma (B16F10) cells [125]. It has been also proved that chalepensin is a mechanism-based inhibitor of cytochrome P450 (CYS) 2A6 [126,127,128]. This is the first report on the antiprotozoal activity against *Entamoeba histolytica* of chalepensin.

### 2.4. Possible Antiprotozoal Mechanism of Action of Carvacrol and Chalepensin

Little knowledge exists about the antiprotozoal mechanism of action of carvacrol but the antimicrobial mechanisms of action of carvacrol have been thoroughly investigated [81,82,83,84,85]. In the following, we describe some facts of these antimicrobial mechanisms which future research might reveal if these apply as well to protozoa. Carvacrol exhibits antimicrobial activity against the biological membranes of bacteria. It exerts its action by rapidly depleting the intracellular ATP pool by reducing ATP synthesis and increasing ATP hydrolysis. Reduction of transmembrane electric potential which is the driving force of ATP synthesis enhances proton permeability of the membrane. At 1 mM carvacrol lowers the internal pH of bacteria from 7.1 to 5.8 according to ion gradients of the cell membrane. Carvacrol (1 mM) decreases cell protein content from 12 mmol/mg to 0.99 mmol/mg by using potassium (K^+^) of bacterial cells in a short time (5 min). Potassium (K^+^) plays a role in the activation of cytoplasmic enzymes, in maintaining osmotic pressure and in the regulation of cytoplasmic pH. Leakage of K^+^ out of the cell is a clear indication of membrane damage. Ultee *et al.* [82] hypothesized a scheme for the mechanism of action of carvacrol through the cytoplasmic membrane of bacteria. According to this hypothesis undissociated carvacrol diffuses through the cytoplasmic membrane and dissociates releasing its proton to the cytoplasm. It then returns undissociated through the membrane into the external environment carrying a potassium ion. Outside the cell carvacrol replaces potassium with a proton and reenters the cell the same way.

The mechanism of action of oregano oils has been shown to be related, especially, to the synthesis of structural components and to the disruption of a series of energy systems. The leakage of ions, ATP and amino acids from bacterial cells explains this phenomenon. Potassium and phosphate ion concentrations were affected at a rate much lower than their MIC values [85].

Carvacrol increases overall permeability of the cytoplasmic membrane by disrupting the outer membranes of Gram negative bacteria leading to the leakage of ATP from the cell. Carvacrol also inhibits ATPase [85]. Similar alterations to those observed in bacteria [85] were also observed on *Giardia lamblia* exposed to essential oils from different sources, especially those where carvacrol had a dominant presence (over 70% of general composition) [28]. The main ultrastructural alterations promoted by essential oils were deformations in typical trophozoite appearance, often roundly shaped, irregular dorsal and ventral surface, presence of membrane blebs, electrodense precipitates in the cytoplasm and nuclei and internalization of flagella and ventral disc. The data suggest that essential oils probably induced cell death by processes associated to the loss of osmoregulation caused by plasmatic membrane alterations [28].

To our knowledge there are no studies regarding the antiprotozoal mechanism of chalepensin but chalepin, a furocoumarin structural related to chalepensin, exerts a potent inhibitory activity against the recombinant enzyme TcGAPDH (glyceraldehyde-3-phosphate dehydrogenase) of *Trypanosoma cruzi* with a strong IC_50_ of 64 µM [129,130]. Further studies are required to establish the antiprotozoal mechanism of carvacrol and chalepensin against *E. histolytica*.

## 3. Experimental Section

### 3.1. General

Melting points were determined on an Electrothermal 9100 apparatus (Electrothermal Engineering Ltd., Southend on Sea, UK). IR spectra were recorded on a Frontier FT-IR spectrometer (PerkinElmer, Waltham, MA, USA) using an ATR accessory. NMR spectra were measured on a Avance DPX 400 Spectrometer (Bruker, Billerica, MA, USA) operating at 400.13 MHz for ^1^H and 100.61 MHz for ^13^C. ESI HR mass spectra were measured on a 4.7 T FT-ICR Mass spectrometer (Bruker, Bremen, Germany). EI MS was recorded on a MAT 95 spectrometer (70 eV, Finnigan, San Jose, CA, USA). TLC was carried out on pre-coated silica gel glass plates 5 cm × 10 cm (Merck silica gel 60 F_254_, Darmstadt, Germany). Normal phase column chromatography was performed on silica gel (60–200 mesh) purchased from J. T. Baker (Phillipsburg, NJ, USA).

### 3.2. Plant Material

The plants were obtained from the field or purchased from Pacalli^®^ (pacalli.com.mx, Monterrey, Mexico). Reference vouchers of the plant material were deposited at the herbarium UNL of the Facultad de Ciencias Biológicas (Universidad Autónoma de Nuevo León). Plant species, botanical name, family, voucher specimens and plant parts used to obtain the extracts are summarized in Table 1. Vegetal material was dried and ground to powder.

### 3.3. Extraction and Isolation

Sixty grams of dried and powdered material from the respective plant was extracted with methanol (MeOH, 600 mL) by using a Soxhlet system for continuous extraction. After filtration, the solvent was evaporated under reduced pressure in a rotary evaporator [25]. The different extracts were conserved in tightly sealed glass vials. The yields are shown in Table 1.

#### 3.3.1. Bioguided Isolation of Carvacrol (**1**)

Ground and dried leaves of *Lippia graveolens* (600 g) were extracted with methanol in several portions of 30 g in a Soxhlet apparatus for 40 h, each charged with 500 mL of CH_3_OH. After filtration of the methanol solutions, the solvent was removed under reduced pressure to yield 260 g of combined extract. The crude extract was analyzed for amebicide activity on trophozoites of *E. histolytica* (HM1:IMSS strain), showing a very high inhibition percentage (89%) by standard concentration of 150 µg/mL. Afterwards, the extract was redissolved in 2 L methanol, being divided in 4 portions of 500 mL each and submitted to liquid-liquid partition with *n*-hexane (500 mL each portion) to yield, after solvent evaporation, 19.9 g of a combined residue with high amebicidal activity (90.9% growth inhibition). The *n*-hexane partition was divided into eighteen portions of ca. 1 g and each of them chromatographed on a silica gel (20 g) column (39 cm × 2 cm) and eluted with stepwise gradients of *n*-hexane–chloroform (100:0, 90:10, 80:20, 70:30, 60:40, 50:50, 40:60, 30:70, 20:80, 10:90, 0:100 *v*/*v*, each 50 mL), chloroform–ethyl acetate (90:10, 80:20, 70:30, 60:40, 50:50, 40:60, 30:70, 20:80, 10:90, 0:100 *v*/*v*, each 50 mL), and finally with 50 mL methanol. A total of 110 subfractions (10 mL) were collected for each column and combined on the basis of their TLC (CHCl_3_–EtOAc, 9:1) profiles into six main fractions as follows: A (subfractions 1–30, 1.1 g), B (subfractions 31–42, 780 mg), C (subfractions 43–63, 7.1 g), D (subfractions 64–72, 2.5 g), E (subfractions 73–75, 1.9 g) and F (subfractions 76–110, 3.6 g). These main fractions, containing the non-polar to the more polar compounds, were used for amebicide assays. Only fraction C showed amebicide activity (82.88% growth inhibition) and was divided into seven portions of ca. 1 g and each of them chromatographed on a silica gel (20 g) column (39 × 2 cm) and eluted with stepwise gradient solvent system consisting of *n*-hexane–chloroform (100:0, 90:10, 80:20, 70:30, 60:40, 50:50, 40:60, 30:70, 20:80, 10:90, 0:100 *v*/*v*, each 50 mL), and finally with 50 mL methanol. A total of 60 subfractions (10 mL) were collected and combined on the basis of their TLC (*n*-hexane–CHCl_3_, 2:8) profiles into three main fractions as follows: G (subfractions 1–27, 1.0 g), H (subfractions 28–38, 4.2 g), I (subfractions 39–60, 677 mg). The amebicide activity was detected on fraction G (44.24% growth inhibition) and very noticeable on fraction H (94.63% growth inhibition); this last was submitted to additional fractionation. Fraction H was divided into four portions of ca. 1 g and each of them later chromatographed on a silica gel (20 g) column (39 cm × 2 cm) and eluted with stepwise gradient solvent system consisting of *n*-hexane–chloroform (100:0, 90:10, 80:20, 70:30, 60:40, 50:50, 40:60, 30:70, 20:80, 10:90, 0:100 *v*/*v*, each 50 mL), and finally with 50 mL methanol. A total of 120 subfractions (5 mL) were collected and combined on the basis of their TLC (*n*-hexane–CHCl_3_, 2:8) profiles into three main fractions as follows: J (subfractions 1–63, 514 mg), K (subfractions 64–90, 1.7 g) and L (subfractions 91–120, 1.0 g). Fraction J (98.4% growth inhibition; IC_50_ 44.30 µg/mL) and Fraction K (95.13% growth inhibition; IC_50_ 44.22 µg/mL) both provided an oil containing a compound with a very intense and characteristic hydrocarbon smell (carvacrol, **1**). Fraction L did not present amebicidal activity.

*Carvacrol* (**1**). Red colored oil; *R_f_* = 0.40 (*n*-hexane–CHCl_3_, 2:8); IR (liquid film) *vmax* (cm^−^^1^): 3392, 3020, 2959, 2927, 2869, 1873, 1720, 1621, 1590, 1523, 1458, 1382, 1363, 1252, 1232, 1173, 863, 810; ^1^H-NMR (400 MHz, CHCl_3_-*d*): δ (ppm) = 7.06 (1H, d, *J* = 7.5 Hz, H-3), 6.74 (1H, d, *J* = 7.5 Hz, H-4), 6.68 (1H, s, H-6), 5.37 (1H, br s, OH), 2.84 (1H, sept, *J* = 6.9 Hz, H-7), 2.24 (3H, s, H-10) 1.24 (6H, d, *J* = 6.9 Hz; H-8, H-9); ^13^C-NMR (100 MHz, CHCl_3_-*d*): δ (ppm) = 153.65 (Cq, C-1), 148.45 (Cq, C-5), 130.83 (CH, C-3), 120.87 (Cq, C-2), 118.76 (CH, C-4), 113.01 (CH, C-6), 33.7 (CH, C-7), 24.02 (2 CH_3_; C-8, C-9), 15.34 (CH_3_, C-10); ESI-MS (+)-mode: *m*/*z* = 323.2 [2M+Na]^+^, 623.4 [4M+Na]^+^; ESI-MS (−)-mode: *m*/*z* = 149.1 [M−H]^−^, 195.1 [M+HCOO]^−^; (calcd. for C_10_H_14_O: 150.21).

#### 3.3.2. Bioguided Isolation of Chalepensin (**2**)

Ground and dried leaves of *Ruta chalepensis* (600 g) were extracted with methanol in several portions of 30 g in a Soxhlet apparatus for 40 h, each charge with 400 mL of CH_3_OH. After filtration of the methanol solutions, the solvent was removed under reduced pressure to yield 124.0 g of combined extract. This extract was analyzed for anti-amoebic activity, showing a 90.5% growth inhibition on trophozoites of *E. histolytica* (HM1:IMSS strain). Afterwards, the extract was redissolved in 1 L of methanol, being divided into four portions of 250 mL each and submitted to liquid-liquid partition with *n*-hexane (750 mL each portion) to yield, after solvent evaporation, 8.2 g of a combined residue with high amebicidal activity (84.66% growth inhibition). The *n*-hexane partition was divided into eight portions of 1 g and each of them chromatographed on a silica gel (22 g) column (30 cm × 2 cm) and eluted with stepwise gradients of *n*-hexane–chloroform (100:0, 90:10, 80:20, 70:30, 60:40, 50:50, 40:60, 30:70, 20:80, 10:90, 0:100 *v*/*v*, each 50 mL), chloroform–ethyl acetate (90:10, 80:20, 70:30, 60:40, 50:50, 40:60, 30:70, 20:80, 10:90, 0:100 *v*/*v*, each 50 mL) and finally with 50 mL methanol. A total of 110 subfractions (10 mL) were collected for each column and combined on the basis of their TLC (CHCl_3_–ethyl acetate, 9.5:0.5) profiles into eight main fractions as follows: A (subfractions 1–22, 328 mg), B (subfractions 23–30, 1197 mg), C (subfractions 31–34, 209 mg), D (subfractions 35–43, 1184 mg), E (subfractions 44–49, 701 mg), F (subfractions 50–55, 649 mg), G (subfractions 56–61, 494 mg), H (subfractions 62–110, 1134 mg). These main fractions, containing the non-polar to the more polar compounds, were used for amebicide assays, resulting in 12.51% growth inhibition for A, 93% for B, 88.26% for C, 89.87% for D, 88.47% for E, 92.05% F, 91.69% for G and 91.06% for H. Fraction B, showing good activity against trophozoites of *E. histolytica* and mainly one compound in TLC with a *R_f_* of 0.41 (*n*-hexane–CHCl_3_, 3:7) was therefore submitted to additional fractionation. Fraction B was chromatographed again on a silica gel (22 g) column (30 cm × 2 cm) using a stepwise gradient solvent system consisting of chloroform–ethyl acetate (100:0, 90:10, 80:20, 70:30 *v*/*v*, each 50 mL), and finally with 50 mL methanol. A total of 50 subfractions (5 mL) were collected, combined on the basis of their TLC (*n*-hexane–CHCl_3_, 3:7) profiles into five main fractions as follows: I (subfractions 1–10, 15 mg), J (subfractions 11–14, 456 mg), K (subfractions 15–24, 466 mg), L (subfractions 25–35, 67 mg), M (subfractions 36–50, 42 mg). Only Fraction J contained the compound with *R_f_* of 0.41 (*n*-hexane–CHCl_3_, 3:7) and was subjected to additional purification on silica gel (22 g) column (30 cm × 2 cm) using a stepwise gradients of chloroform–ethyl acetate (100:0, 90:10 *v*/*v*, each 200 mL). A total of 80 subfractions (5 mL) were collected, combined on the basis of their TLC (*n*-hexane–CHCl_3_, 3:7) profiles into four main fractions as follows: N (subfractions 1–11, 17 mg), O (subfractions 12–17, 243 mg), P (subfractions 18–44, 92 mg), Q (subfractions 45–80, 18 mg). Fraction O produced pure chalepensin (**2**), showing 98.4% growth inhibition against *E. histolytica* and an IC_50_ value of 45.95 µg/mL. Fraction P was subjected to additional purification on a silica gel (22 g) column (30 cm × 2 cm) using stepwise gradients of chloroform–ethyl acetate (100:0, 95:05, *v*/*v*, each 150 mL) and finally 50 mL of methanol as eluent. A total of 70 subfractions (5 mL) were collected, combined on the basis of their TLC (*n*-hexane–CHCl_3_, 3:7) profiles into three main fractions as follows: R (subfractions 1–27, 37 mg), S (subfractions 28–47, 24 mg) and T (subfractions 48–70, 8 mg). Fraction R rendered additional pure chalepensin.

*Chalepensin* (**2**). Colorless needles; M.p. 75 °C; *R_f_* = 0.41 (*n*-hexane–CHCl_3_, 3:7); IR (powder) *vmax* (cm^−^^1^): 3016, 2968, 2932,1715, 1629, 1583, 1542, 1451, 1024, 751; ^1^H-NMR (400 MHz, CDCl_3_-*d*): δ (ppm) = 7.68 (1H, s, H-4), 7.67 (1H, d, *J* = 2.24 Hz, H-2’), 7.65 (1H, s, H-5), 7.43 (1H, s, H-8), 6.82 (1H, dd, *J* = 2.24, 0.96 Hz, H-3’), 6.21 (1H, dd, *J* = 17.2, 11.0 Hz, H-12), 5.10 (1H, s, H-13a-*cis*), 5.07 (1H, dd, *J* = 8.16, 0.97 Hz, H-13b-*trans*), 1.52 (6H, s, 14-CH_3_, 5-CH_3_); ^13^C-NMR (100 MHz, CDCl_3_-*d*): δ (ppm) = 159.94 (C_q_, C-2), 155.84 (C_q_, C-7), 151.28 (C_q_, C-9), 146.59 (CH, C-2’), 145.45 (CH, C-12), 138.34 (CH, C-4), 133.09 (C_q_, C-3) 124.57 (C_q_, C-6), 119.52 (CH, C-5), 115.91 (C_q_, C-10), 112.33 (CH_2_, C-13), 106.35 (CH, C-1'), 98.95 (CH, C-8), 40.51 (C_q_, C-11), 26.15 (2 CH_3_, C-14, C-15); ESI-MS (+)-mode: *m*/*z* = 255.1 [M+H]^+^, 277.1 [M+Na]^+^, 531.2 [2M+Na]^+^; (+)-ESI HR MS: *m*/*z* = 255.1014 [M+H]^+^ (calcd. For C_16_H_15_O_3_: 255.1016), 277.0835 [M+Na]^+^ (calcd. for C_16_H_14_NaO_3_: 277.0835).

### 3.4. Antiprotozoal Assay

#### 3.4.1. Test Microorganisms

*Entamoeba histolytica strain* HM-1:IMSS was obtained from the microorganism culture collection of the Centro de Investigación Biomédica del Noreste (CIBIN-IMSS) in Nuevo León, Mexico. The trophozoites were grown axenically and maintained in peptone, pancreas and liver extract plus bovine serum [131]. The trophozoites were employed at log phase of growth (2 × 10^4^ cells/mL) by all the performed bioassays [132,133].

#### 3.4.2. *In Vitro* Assay for *Entamoeba histolytica*

The MeOH extract from each plant was dissolved in DMSO and adjusted to a concentration of 150 µg/mL in a suspension of *E. histolytica* trophozoites at logarithmic phase in PEHPS medium containing 10% of bovine serum. Vials were incubated for 72 h, then chilled in iced water for 20 min and the number of dead trophozoites per milliliter was counted by using a hemocytometer. Each extract assay was performed by triplicate [132,133]. Each test included a positive control by using metronidazole and a negative control by using *E. histolytica* suspension in PEHPS medium with no extract added. The inhibition percentage was estimated as the number of dead cells compared with the untreated controls.

The same procedure was performed with fractions or pure isolated compounds.

#### 3.4.3. *In Vitro* IC_50_ Determination

The MeOH extract from each plant was dissolved in DMSO and adjusted to 150, 75, 32.5 and 16.25 µg/mL with a suspension of *E. histolytica* trophozoites at logarithmic phase in PEHPS medium containing 10% of bovine serum. Vials were incubated for 72 h, then chilled in iced water for 20 min and the number of dead trophozoites per milliliter was determined by using a hemocytometer. Each extract assay was performed by triplicate. The 50% inhibitory (IC_50_) concentration of each extract was determined by using a Probit analysis with a 95% confidence level. The same procedure was performed with fractions or pure isolated compounds.

## 4. Conclusions

*Entamoeba histolytica* is the most common parasite to cause enteric protozoan infections. The drug of choice used to treat amoebic dysentery is metronidazole, which has been associated with unpleasant side effects [134,135,136], therefore alternative drugs are needed and medicinal plants may be an important alternative source of new antiamoebic compounds. The results of the antiprotozoal screening in this work support the popular uses of 18 of the studied species for the treatment of diarrhea and dysentery in Mexican traditional medicine. The extracts from both *Lippia graveolens* Kunth and *Ruta chalepensis* Pers showed the most significant antiprotozoal activity and were submitted to a bioguided fractionation. Structure elucidation of the isolated compounds was accomplished by spectroscopic and mass spectrometric data. The methanolic extract of *L. graveolens* rendered carvacrol (**1**) with 95%–98% inhibition against *E. histolytica* at a concentration of 150 µg/mL (IC_50_ 44.3 µg/mL) and from the methanolic extract of *R. chalepensis* chalepensin (**2**) with 98.4% inhibition at a concentration of 150 µg/mL (IC_50_ 45.95 µg/mL) was recovered. To our knowledge, this is the first report on the antiamoebic activity of carvacrol and chalepensin, both known compounds with other notable pharmacological activities. These compounds may also offer new opportunities for treating amoebiasis and other important and often neglected diseases [137] or be useful as lead compounds in the development of new antiprotozoal agents. Further work for isolation of other active constituents from these plants is under way.

## Supplementary Materials

Supplementary materials can be accessed at: http://www.mdpi.com/1420-3049/19/12/21044/s1.
