# Supplementary material for: Antiprotozoal Activity against Entamoeba histolytica of Plants Used in Northeast Mexican Traditional Medicine. Bioactive Compounds from Lippia graveolens and Ruta chalepensis
Source: Molecules. 2014 Dec 15;19(12):21044–65. doi: 10.3390/molecules191221044 (PMC6271573; doi:10.3390/molecules191221044)

Supplementary Materials

Figure S1. ^{1}H-NMR Spectrum of Carvacrol (**1**) $CDCl_3$, 400 MHz).

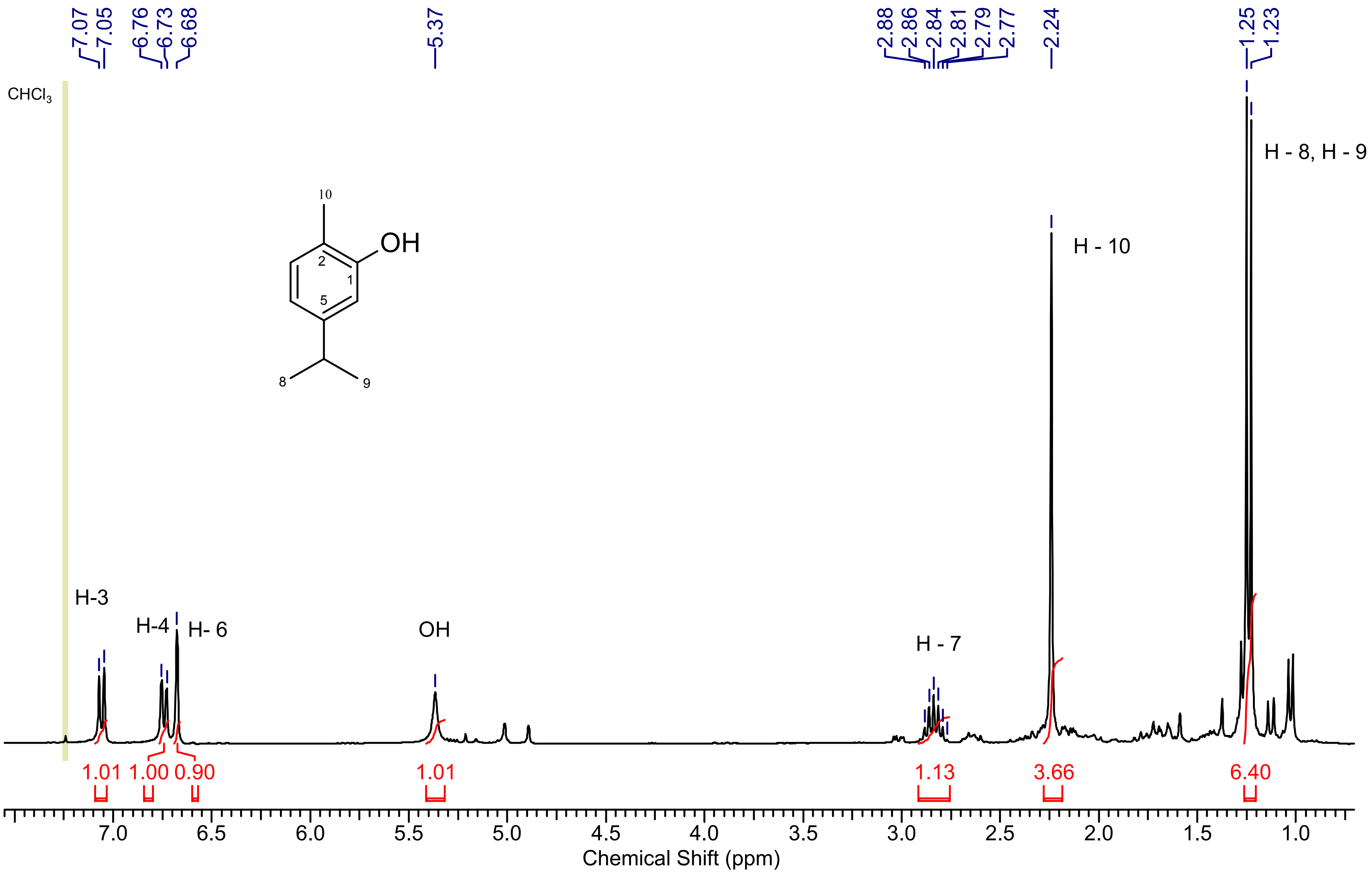

Figure S2. ^{13}C-NMR Spectrum of Carvacrol (**1**) ($CDCl_3$, 100 MHz).

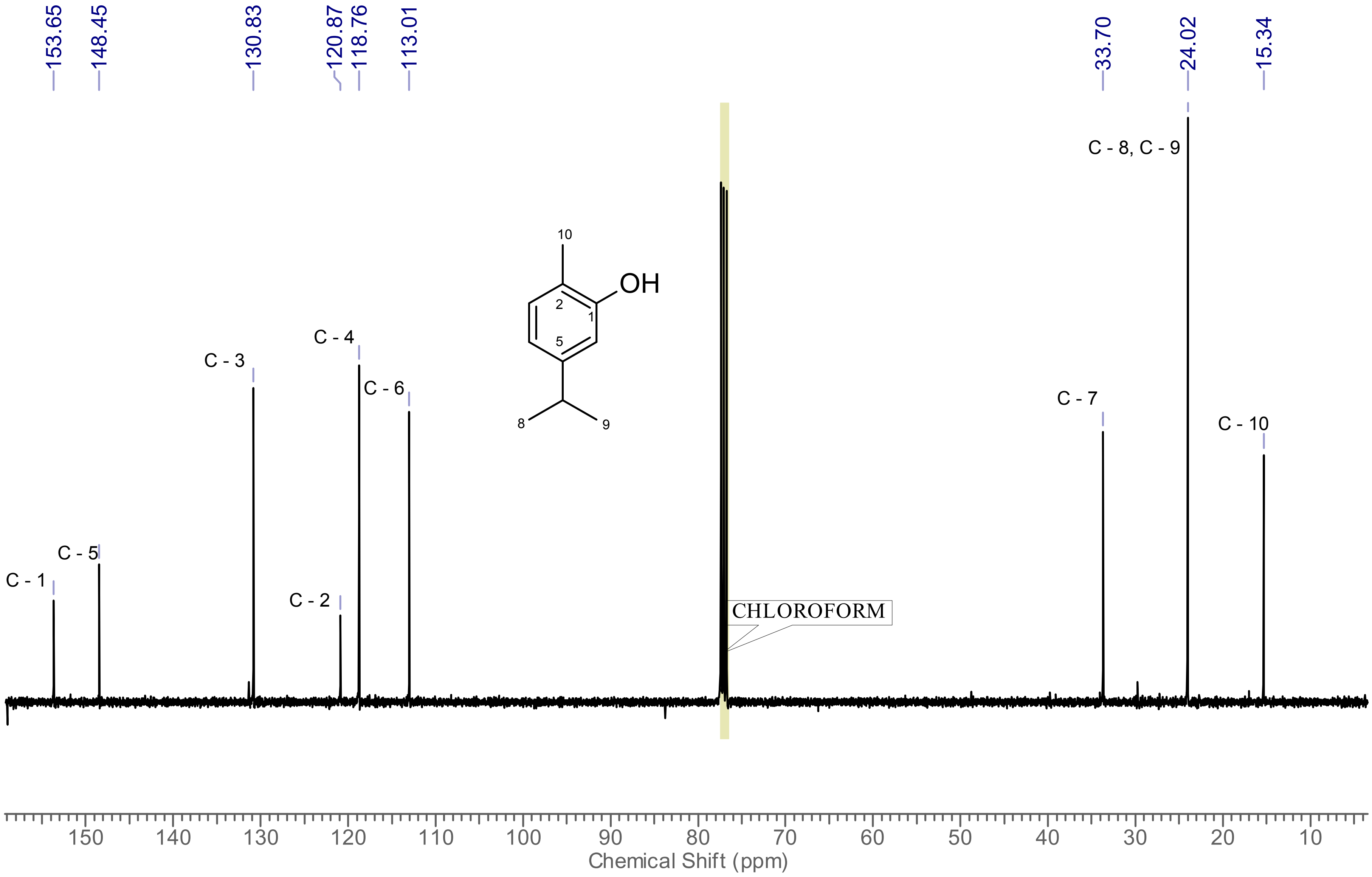

Figure S3. ATP ^{13}C-NMR Spectrum of Carvacrol ($CDCl_3$, 100 MHz).

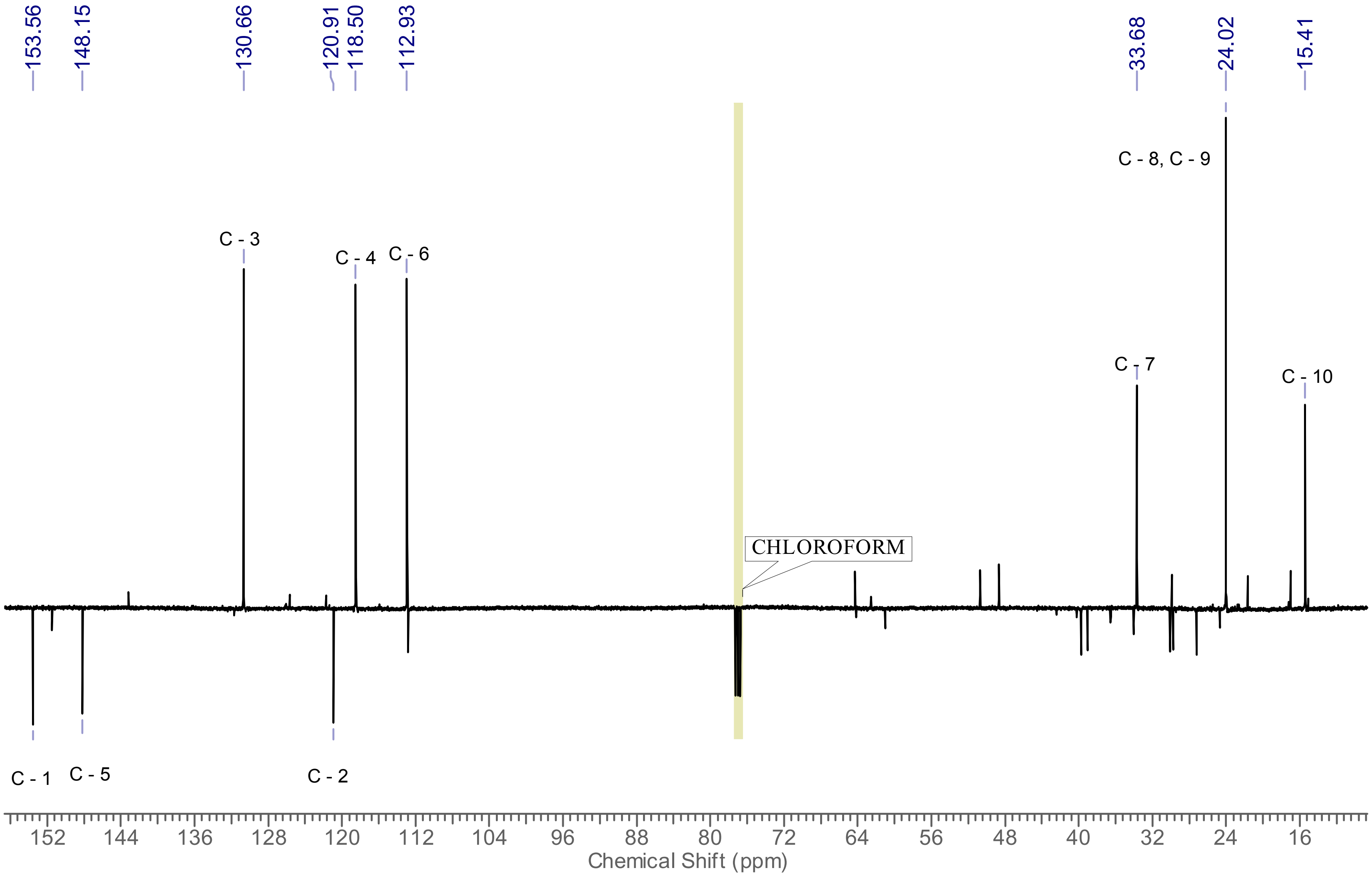

Figure S4. COSY Spectrum of Carvacrol (**1**) ($CDCl_3$, 400 MHz).

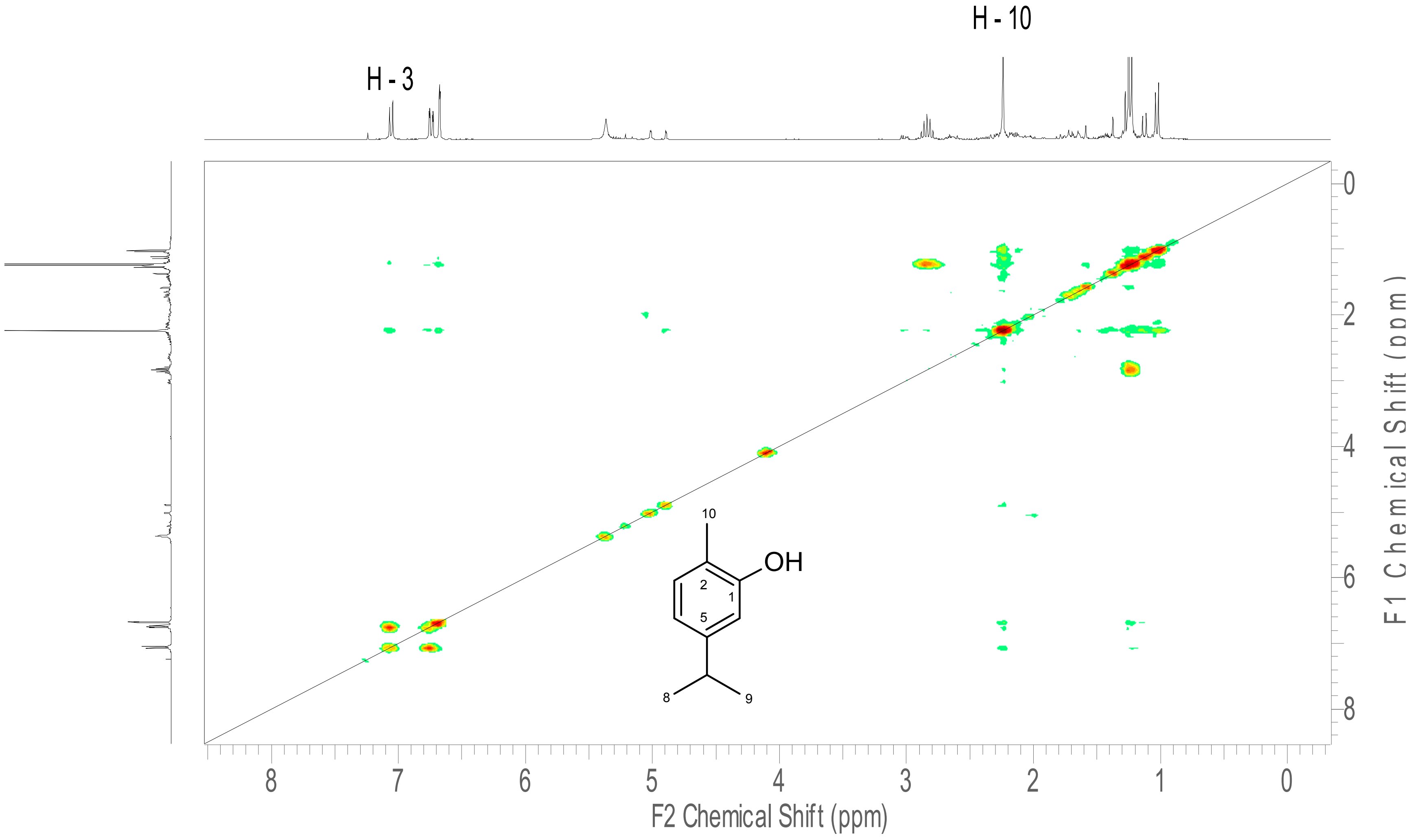

Figure S5. ^{1}H-NMR spectrum of Chalepensin (**2**) ($CDCl_3$, 400 MHz).

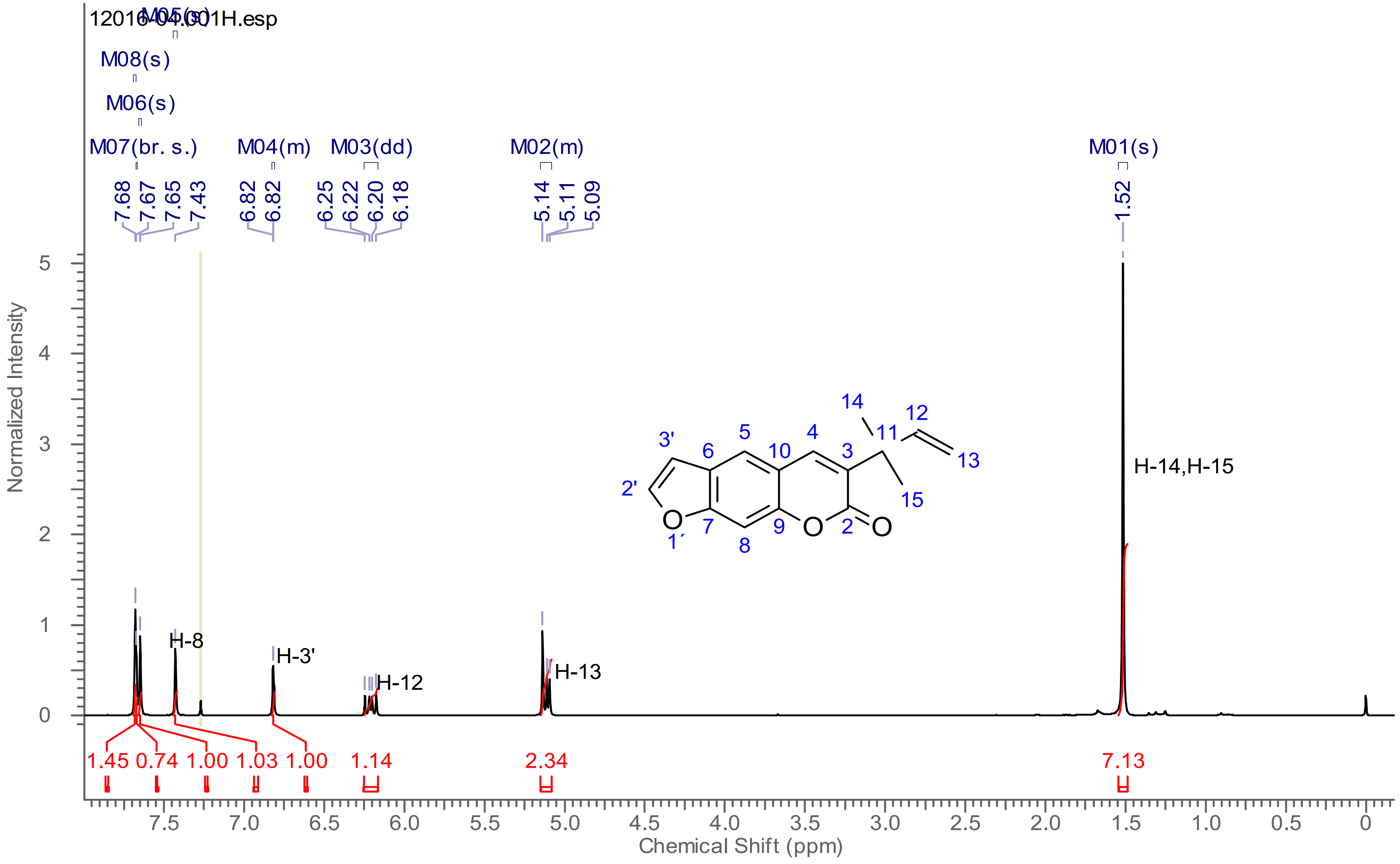

Figure S6. ^{1}H-NMR spectrum of Chalepensin (**2**) ($CDCl_3$, 400 MHz).

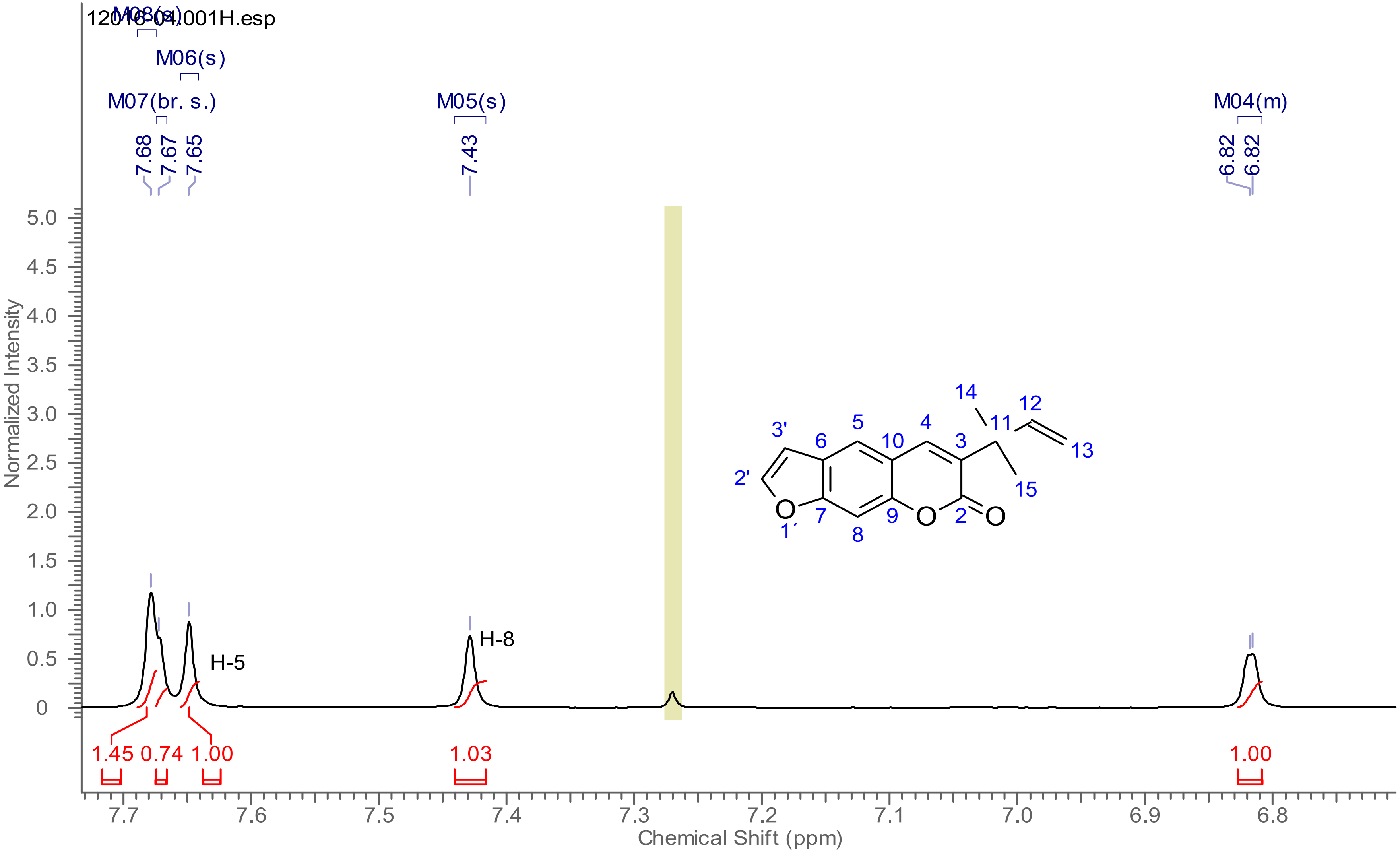

Figure S7. ¹H-NMR spectrum of Chalepensin (**2**) ($CDCl_3$, 400 MHz).

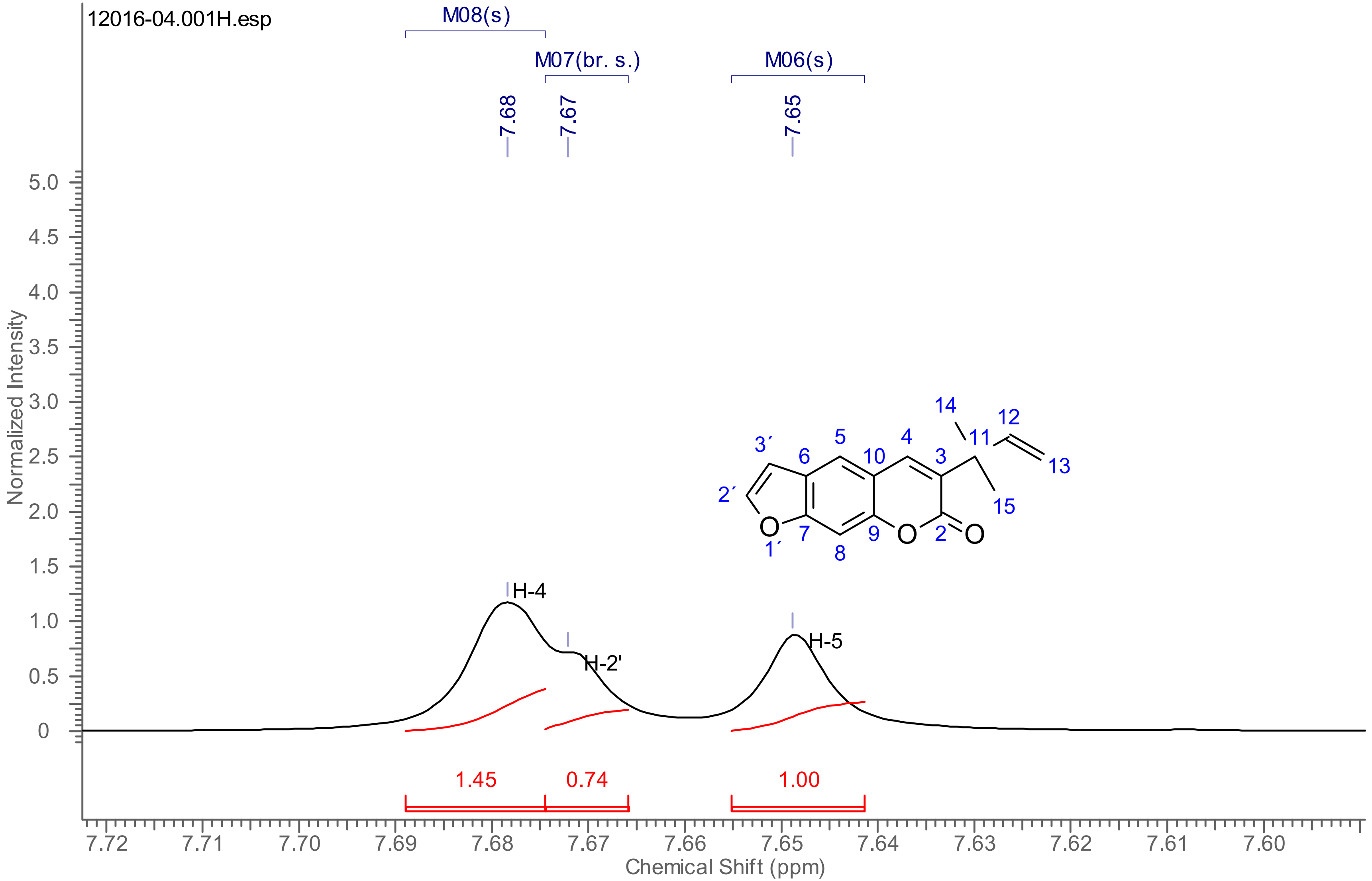

Figure S8. ^{13}C-NMR spectrum of Chalepensin (**2**) ($CDCl_3$, 100 MHz).

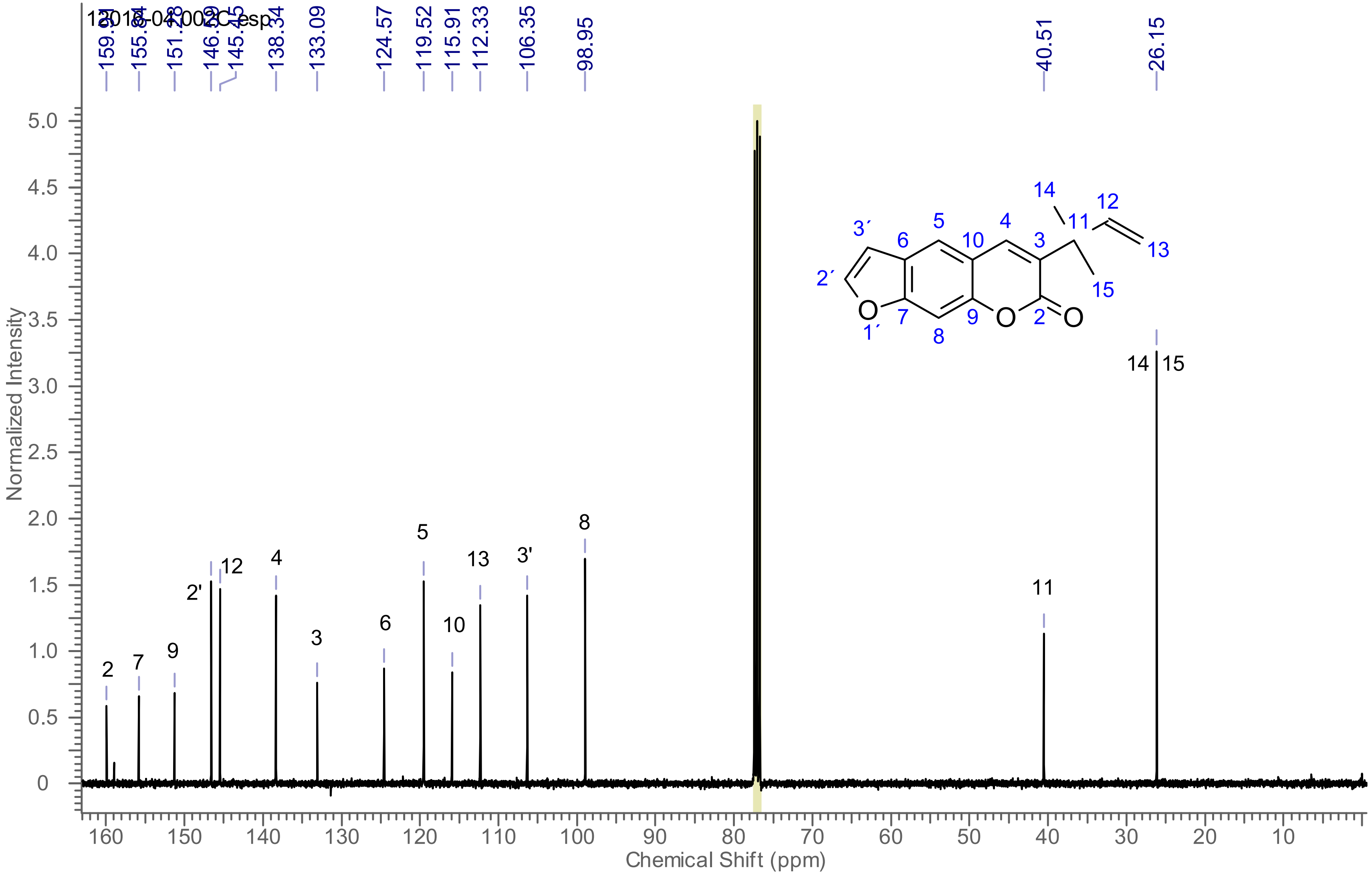

Figure S9. DEPT-135 spectrum of Chalepensin (**2**) ($CDCl_3$, 100 MHz).

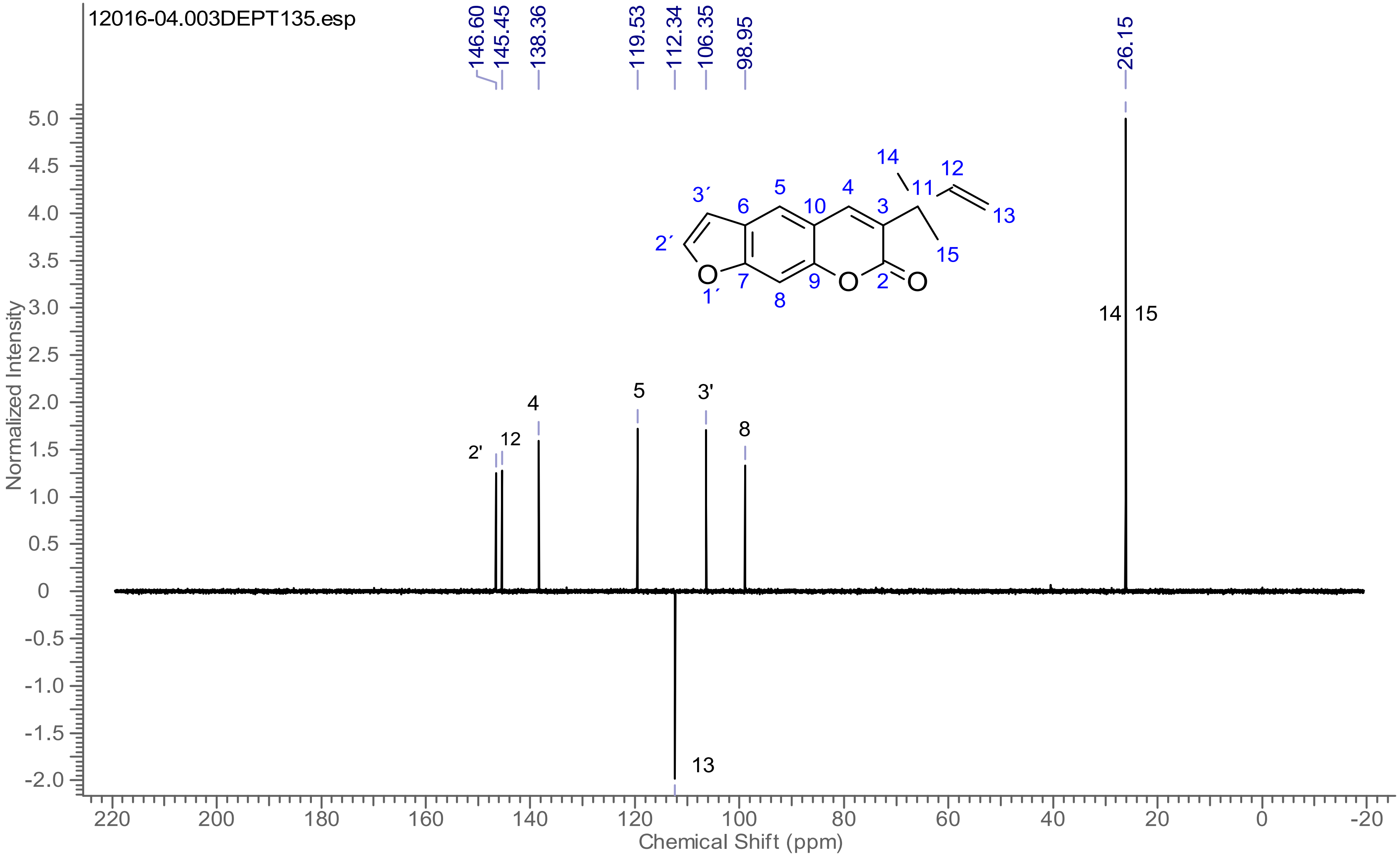

Figure S10. ^{1}H-^{1}H COSY spectrum of Chalepensin (**2**) ($CDCl_3$, 400 MHz).

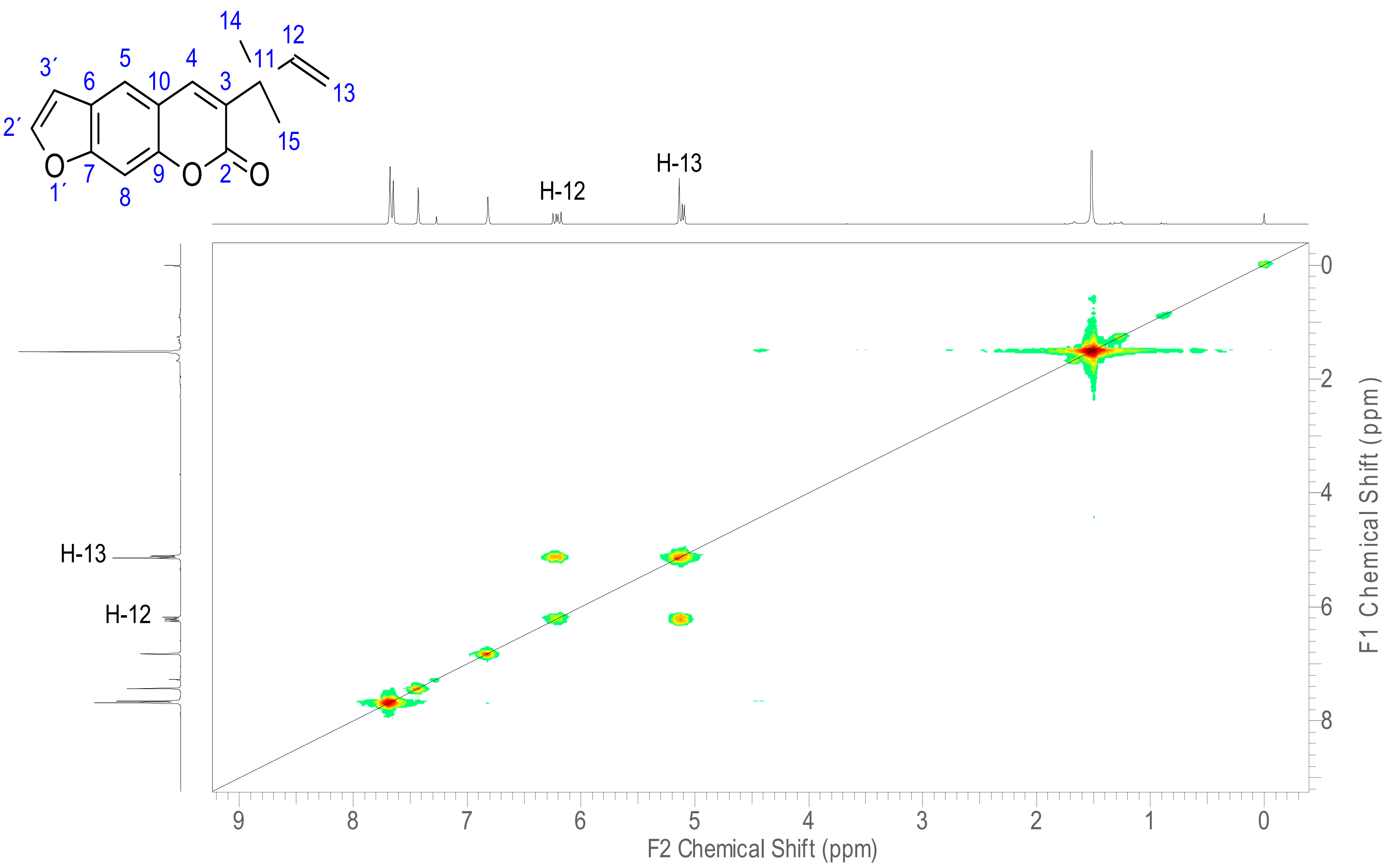

Figure S11. ^{1}H-^{13}C-HSQC spectrum of Chalepensin (**2**) ($CDCl_3$, 100 MHz).

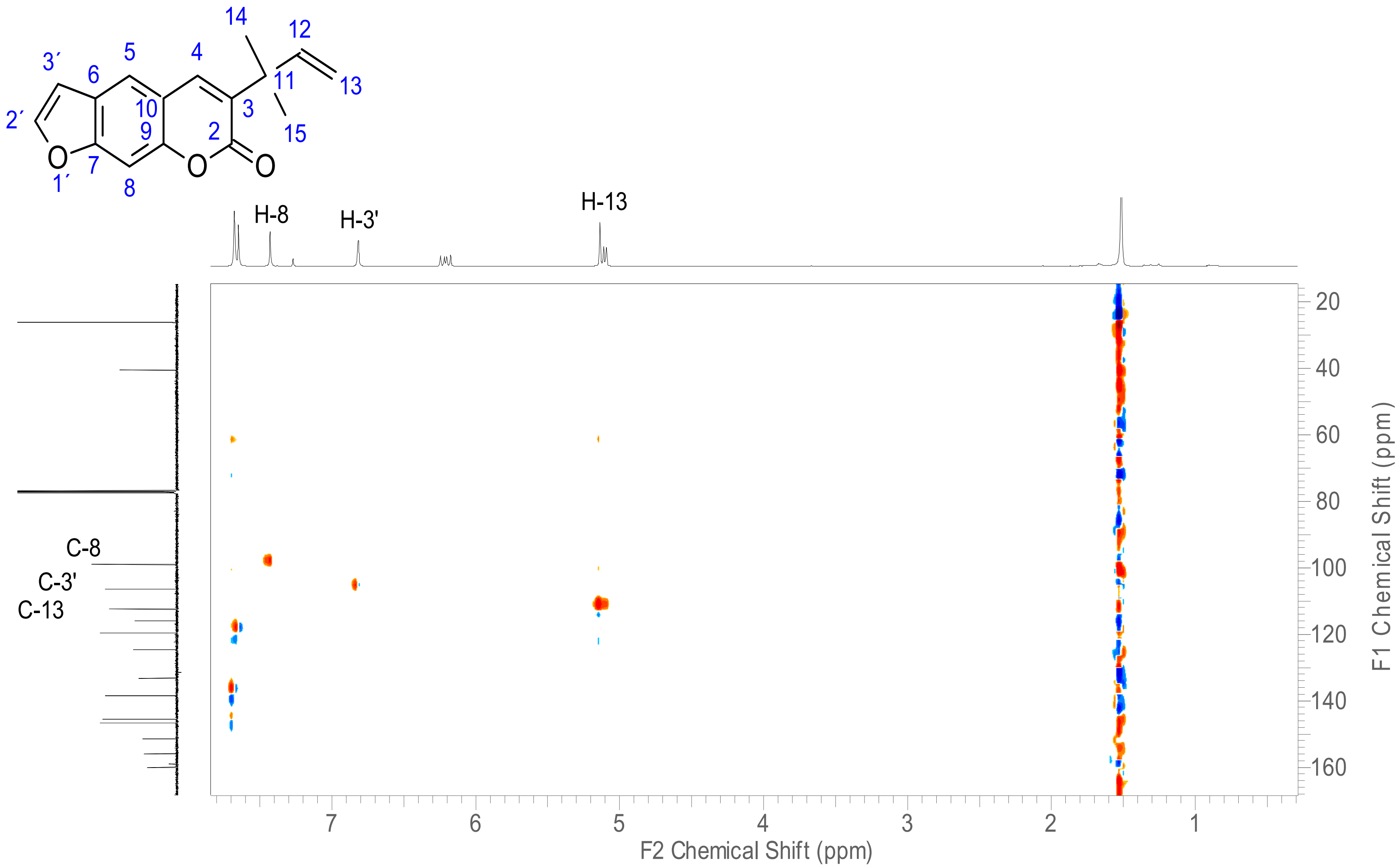

Figure S12. ^{1}H-^{13}C-HSQC spectrum of Chalepensin (**2**) ($CDCl_3$, 100 MHz).

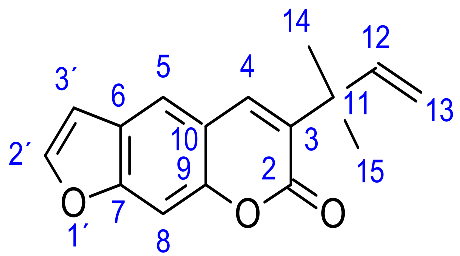

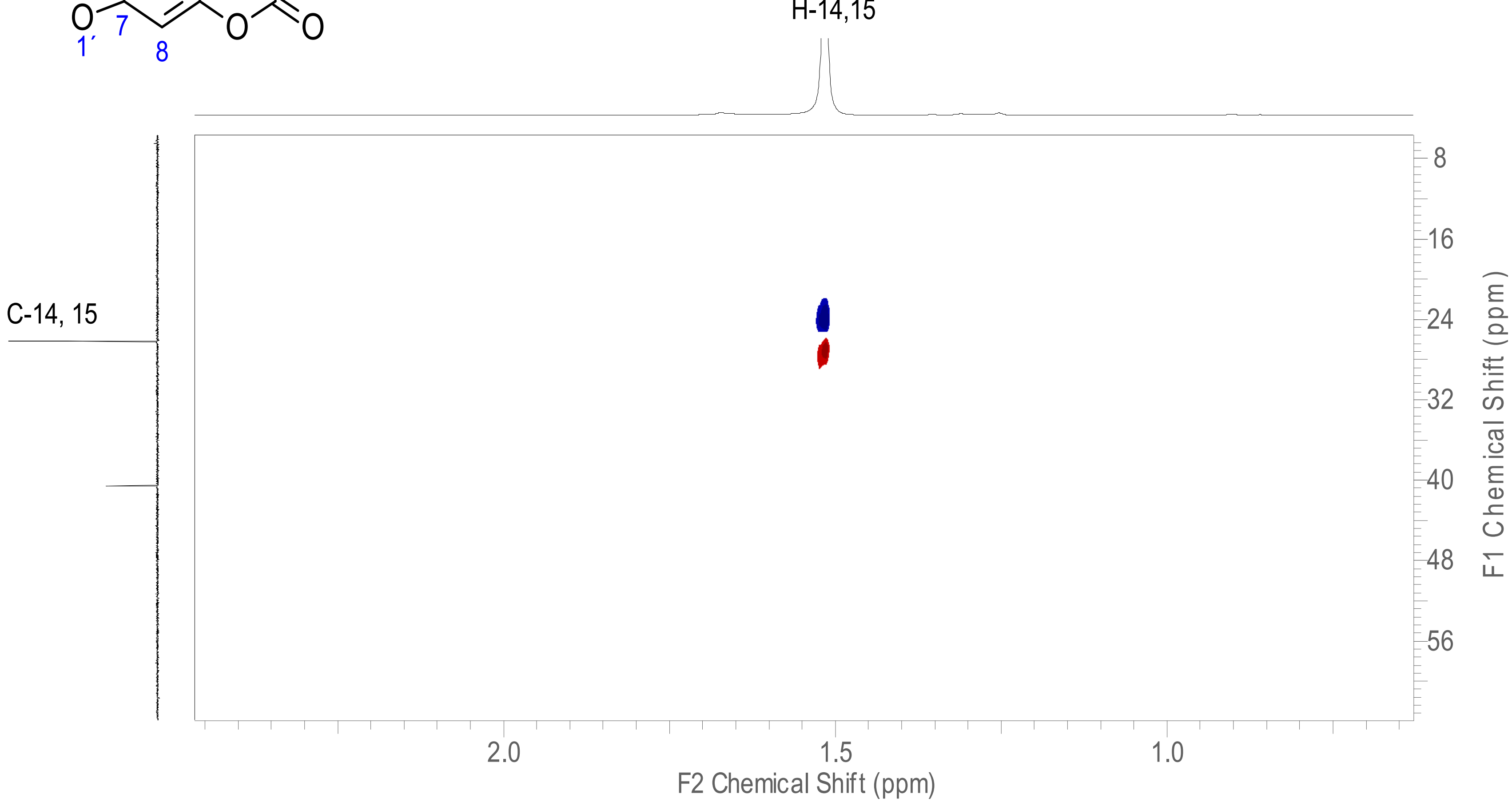

Figure S13. ^{1}H-^{13}C-HSQC spectrum of Chalepensin (**2**) ($CDCl_3$, 100 MHz).

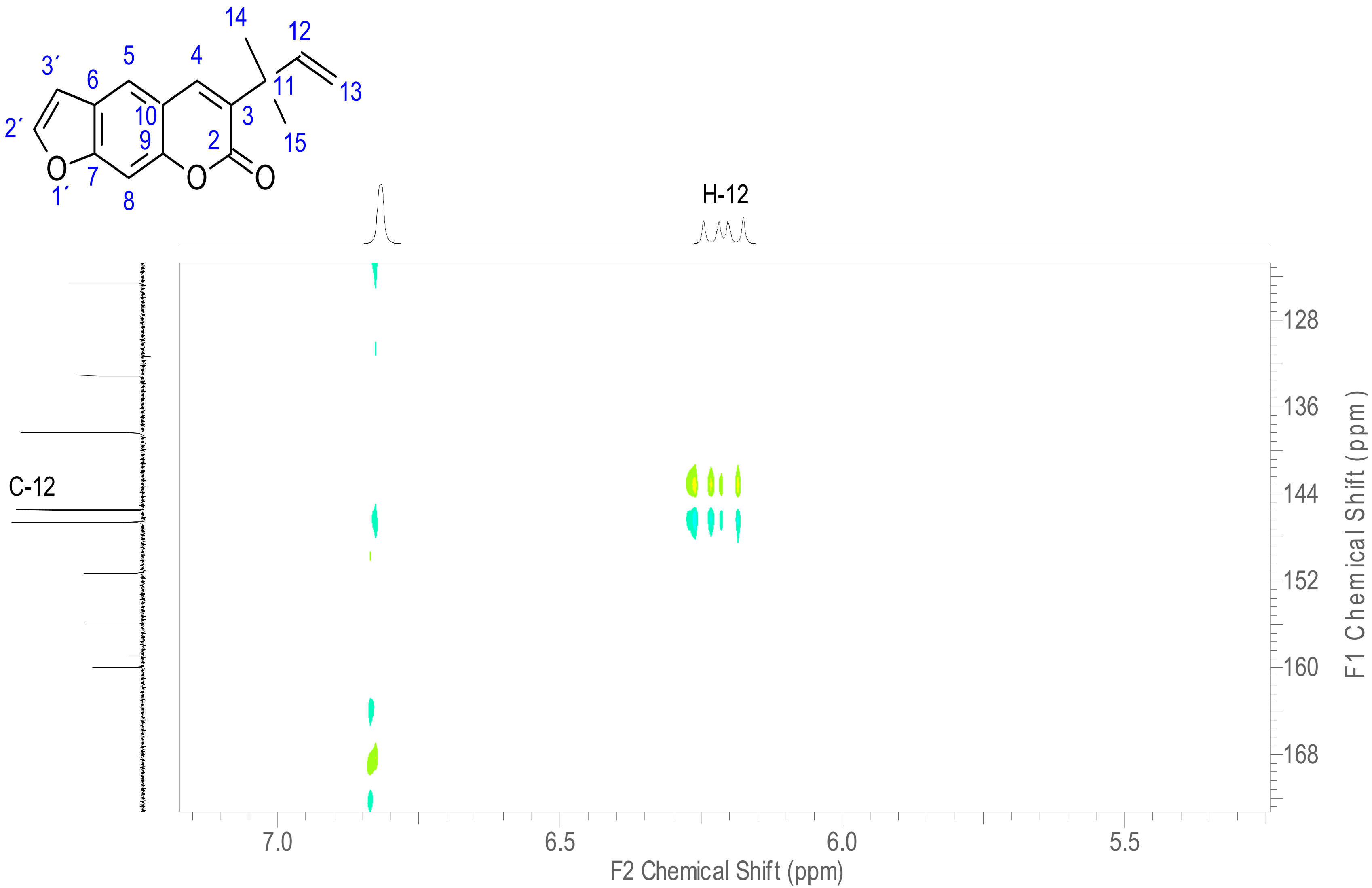

Figure S14. ^{1}H-^{13}C-HMBC spectrum of Chalepensin (**2**) ($CDCl_3$, 100 MHz).

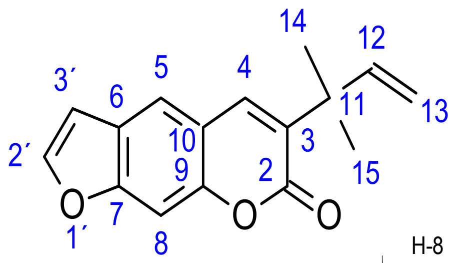

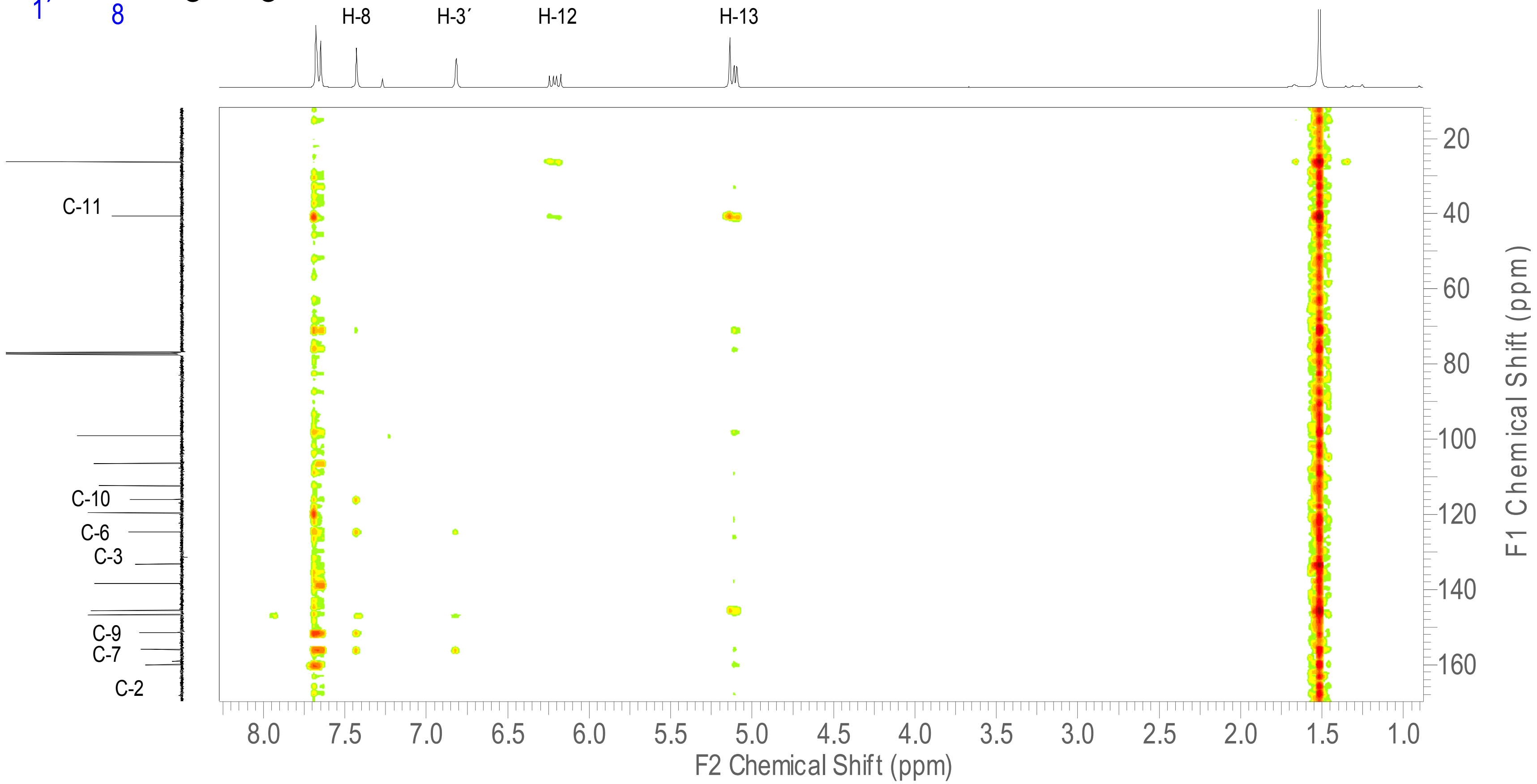

Figure S15. ^1H-^1H-NOESY spectrum of Chalepensin (**2**) ($CDCl_3$, 400 MHz).

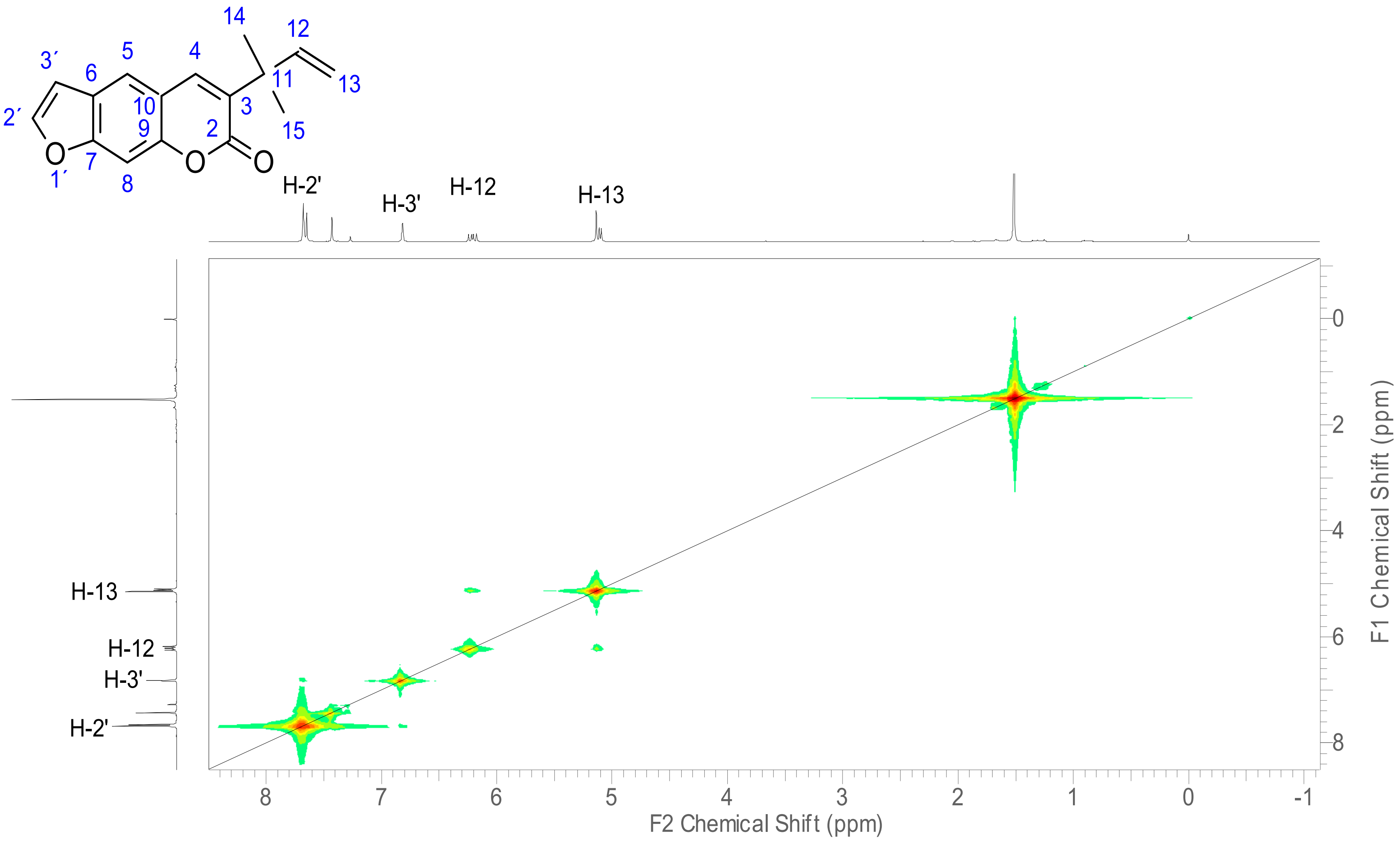

Supplement: Supplementary file 1 [file molecules-19-21044-s001.pdf]
